# Functional block in the initiation and maintenance of common flutter: detailed electrophysiological study and electro-anatomical mapping

**DOI:** 10.3389/fcvm.2024.1494836

**Published:** 2024-11-15

**Authors:** Marine Arnaud, Benjamin Sacristan, Meleze Hocini, Pierre Jais, Michel Haissaguerre, Josselin Duchateau

**Affiliations:** ^1^ Department of Cardiac Pacing and Electrophysiology, Hopital Cardiologique du Haut-Leveque, Bordeaux University Hospital (CHU), Bordeaux, France; ^2^ IHU Liryc, Electrophysiology and Heart Modeling Institute, University Bordeaux, Bordeaux, France

**Keywords:** common flutter, initiation, maintenance, mechanisms, pathophysiology

## Abstract

**Introduction:**

The precise pathophysiology of common atrial flutter remains imperfectly known. The mechanisms of arrhythmia initiation and the role of areas of slow conducting myocardium and functional block are still debated topics.

**Methods:**

We conducted a detailed electrophysiological study of a patient to illustrate and refine these concepts. Prior to CTI ablation, electrophysiological study and electro-anatomical mapping were performed, focusing on initiation and maintenance mechanisms of the arrhythmia.

**Results:**

The initiation of common atrial flutter takes place on the septal aspect of the cavo-tricuspid isthmus where functional unidirectional conduction block occurs. The direction of activation is therefore frequently counter-clockwise, and the arrhythmia stabilizes around the vena cavas and sinus venosus/crista terminalis region. No conduction slowing is present.

**Conclusions:**

Common atrial flutter initiates when functional unidirectional conduction block occurs on the septal cavotricuspid isthmus. Its rotation is limited by anatomical and functional boundaries.

## Introduction

Common atrial flutter refers to a macro-reentrant tachycardia around the tricuspid valve, in the clockwise or counter-clockwise direction (1). This arrhythmia is the most common organized atrial tachycardia and—as such—its initiation and maintenance have been thoroughly studied (2, 3). In terms of initiation, clinical studies have demonstrated the importance of premature atrial contractions (4) (PACs), and their role in determining the reentry direction (5). In terms of maintenance, the arrhythmia rotates around the tricuspid valve, and channels through the narrow region between this valve and the inferior vena cava called the cavo-tricuspid isthmus (CTI). Strategies targeting the initiation of the arrhythmia (pulmonary vein isolation to avoid premature atrial contractions) (6) or its maintenance (linear ablation at the CTI) have both demonstrated their efficiency, reinforcing our confidence in these two pathophysiological components.

Despite this, the precise pathophysiology of this arrhythmia remains imperfectly understood. More specifically, the precise mechanisms of arrhythmia initiation and the role of areas of slow conducting myocardium and functional block are still debated topics.

In this work, we examine existing literature describing the role of functional block in the initiation and maintenance of common atrial flutter, and a detailed electrophysiological study of a patient to illustrate and refine these concepts.

## Methods

### Mapping procedure

The initiation and maintenance mechanisms of common atrial flutter were studied in 10 patients in total. Prior to CTI ablation, electrophysiological study and electro-anatomical mapping were performed, focusing on initiation and maintenance mechanisms of the arrhythmia. Electro-anatomical mapping was performed using the Carto3 mapping system (Biosense Webster, Diamond Bar, CA). A steerable quadripolar catheter (Dynamic XT, Boston Scientific, MA) was positioned in the coronary sinus and used to perform atrial pacing. A multipolar mapping catheter (Octaray, Biosense Webster, Diamond Bar, CA) was used to perform the right atrial anatomical shell reconstruction and recording cardiac electrograms during pacing maneuvers. During pacing, it was positioned on the CTI, and the pacing protocol described hereafter was performed. In all but one patient, conduction block led to immediate initiation of CTI-dependent flutter, precluding thorough mapping of the arrhythmia onset.

The patient presented in this study is a 53-year old male patient with no comorbidities who was scheduled for atrial flutter ablation due to recurrent episodes of symptomatic counterclockwise common atrial flutter. The procedure was carried out under conscious sedation.

## Results

### Functional block identification and flutter induction protocol

Based on prior studies (5, 7, 8) functional block leading to atrial flutter initiation was presumed to occur in the “septal isthmus” region, and at the junction of the sinus venosus and crista terminalis. We therefore performed an atrial stimulation study to identify these functional blocks. Programmed atrial stimulation with S1–S2 was performed by pacing the proximal coronary sinus, starting at 600-400 ms intervals with 10 ms decrement up to the atrial effective refractory period (ERP). The multipolar mapping catheter was positioned on the CTI to identify abrupt changes in activation sequence or timing, reflecting functional block. Since S2 extrastimuli failed to produce functional block at atrial ERP (280 ms), S3 stimuli were added, starting at 300 ms, with 10 ms decrements with fixed S2 at 300 ms.

An abrupt change in the activation pattern on the CTI occurred after a 600-300-240 ms sequence. This pacing sequence was then repeated to construct a right atrial map of the S1, S2 and S3 activation patterns (see below).

**Figure 1:**
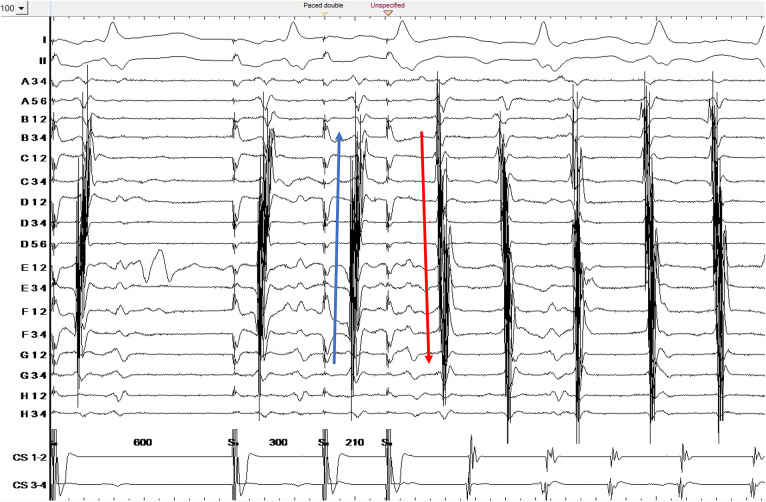
Counterclockwise common atrial flutter induction. Ortaray is positioned at the cavo-tricuspid isthmus level. Note the change in electrogram activation sequence for S3 due to functional block in the septal isthmus region. S3 depicts a unidirectional conduction block on the septal cavo-tricuspid isthmus.

When the map was complete, the S3 beat was further decremented. No additional abrupt changes were observed, until a sequence of 600-300-210 ms at which point CTI-dependent flutter was induced (Figure 1). A map of the flutter was created and compared to the previous maps.

### S1, S2 and S3 mapping technique and interpretation

**Figure 2:**
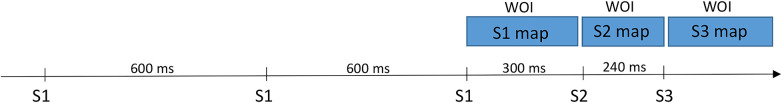
Programmed atrial stimulation with S1-S2-S3 trains (from the proximal coronary sinus). In order to obtain S1, S2 and S3 maps during a single acquisition, the window of interest (WOI) was first set to register S3 potentials, then moved backwards to encompass S2 potentials and finally moved back again to bracket S1 potentials.

To map the S1, S2 and S3 activation patterns, Carto acquisition filters were set to acquire beats corresponding to cycle lengths in the 235–245 ms range on the coronary sinus, allowing us to construct an S3 activation map. All other filters were turned off except for mapping catheter position stability. The map was then copied, and the window of interest changed to visualize the S2 beat at the same points, and finally changed again to image the last S1 beat of the drive train (Figure 2).

**Figure 3:**
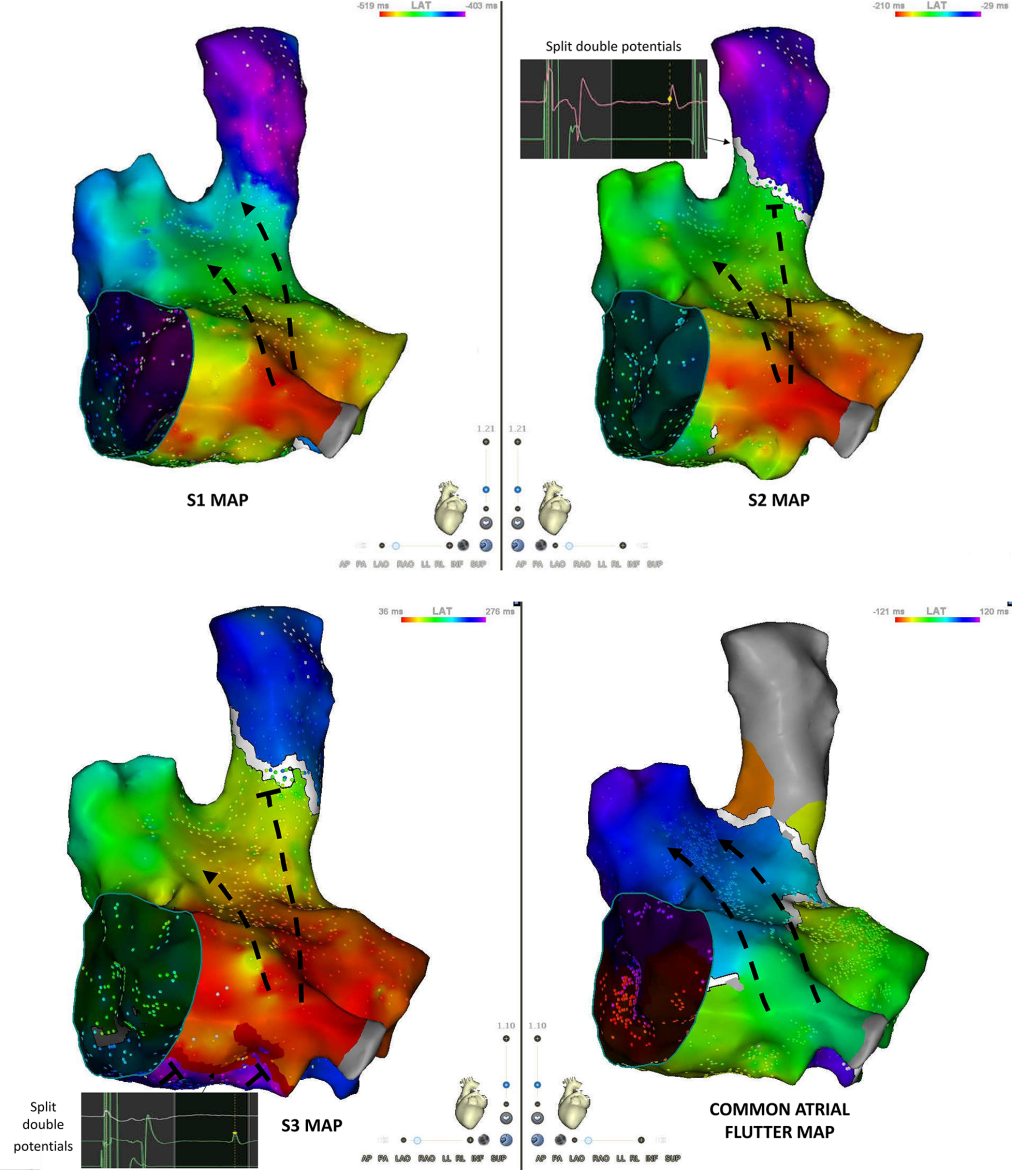
S1, S2, S3 and flutter maps of the right atrium in an anteroseptal view. S2 is associated with the occurrence of a conduction block at the junction between the superior vena cava and the right atrium. Double potentials are recorded along the line of block. S3 maps shows the appearance of a line of block in the “septal isthmus” region with double potentials. Atrial flutter map shows a counter-clockwise activation sequence.

The main qualitative difference between the S1 and S2 maps is the appearance of a line block at the septal aspect of the SVC-RA junction (Figures 3A,B). This line of block is not relevant for CTI-dependent flutter induction.

The S3 map shows the additional appearance of a line of block at the septal aspect of the cavo-tricuspid isthmus (Figures 3B,C). The electrogram activation sequence inversion on the Octaray positioned at the CTI level clearly depicts this phenomenon (Figure 1).

**Figure 4:**
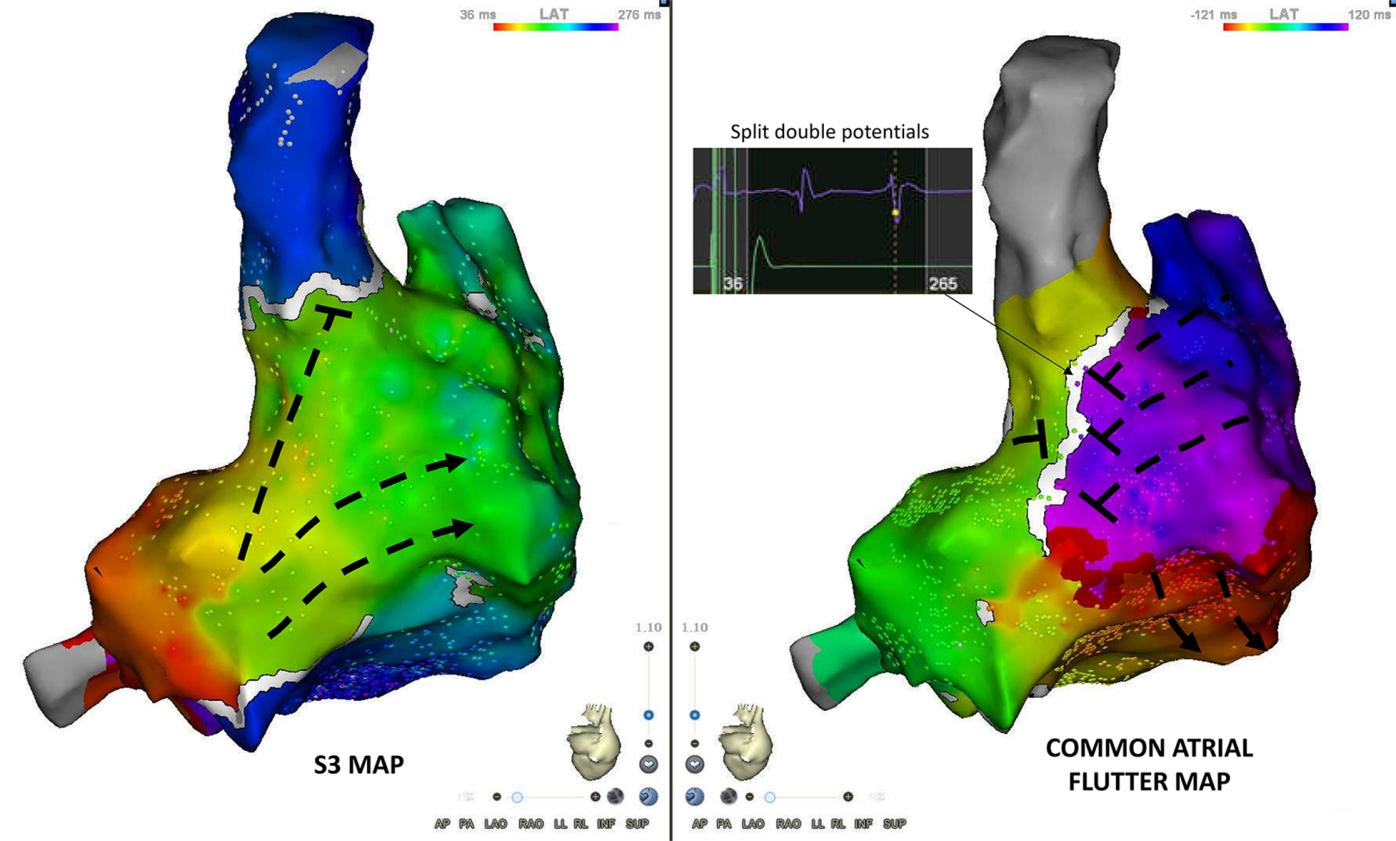
Posterior view of S3 and flutter maps. The main qualitative difference between both maps is the appearance of conduction block along the posterior aspect of the right atrium (sinus venosus/crista terminalis region). Double potentials are recorded along the line of block.

The main qualitative difference between the atrial flutter and S3 maps was the line of block along the posterior aspect of the right atrium in the sinus venosus/crista terminalis region on which double potentials could be recorded (Figure 4). Isochronal mapping showed no significant conduction slowing in other areas.

## Discussion

Through this report, we illustrate the role of functional block in the initiation and maintenance of common atrial flutter.

### Initiation: unidirectional conduction block on the septal cavo-tricuspid isthmus

In this patient, a double extra-stimulus provoked a unidirectional conduction block on the septal aspect of the cavo-tricuspid isthmus.

We confirm the observation made by Cosio et al. Indeed, the authors did not have electroanatomic maps at the time but based on electrogram analysis they described that typical flutter begins by low septal block (7). Olgin et al., using fluoroscopy and electrogram analysis described the site of unidirectional block during the initiation of clockwise and counterclockwise flutter was in the low right atrium isthmus (8).

Conduction block on the septal aspect of the cavo-tricuspid isthmus may be due to the occurrence of source-sink mismatch in this region characterized by a specific fiber arrangement. Atrial myocardium is very thin and fiber directions criss-cross in the approaches to the atrioventricular node (9), without the circumferential fibers that follow the tricuspid ring (10).

Moreover, the septal aspect of the cavotricuspid isthmus is consistently a site of wavefront collision in sinus rhythm (11). This fact may favor the occurrence of functional conduction block when a PAC from another location associated with a different wavefront occurs.

The direction of the circuit depends on the orientation of the conduction block. We describe that left sided activation (extrastimuli from the coronay sinus) induces counterclockwise flutter. Likewise, Olgin et al. showed that pacing from the smooth right atrium induced counterclockwise flutter, whereas pacing from the trabeculated right atrium induced clockwise flutter (8). PACs originating from the pulmonary veins are the main triggers for common flutter (6). Therefore, the conduction is directed from septal to lateral isthmus and unidirectional block induces counter-clockwise rotation. The latter type of flutter is thus the most prevalent.

### Maintenance: circuit rotating around three anatomical and a functional boundary

After induction, the circuit stabilizes with a counter-clockwise rotation around three anatomical obstacles: the tricuspid annulus, the inferior and superior vena cava ostia.

Another conduction block is necessary for common atrial flutter maintenance: functional block along the posterior aspect of the right atrium, in the sinus venosus/crista terminalis region.

This phenomenon has been described by Friedman et al. in both clockwise and counterclockwise atrial flutter (12). It is probably due to craniocaudal alignment of myocardium at the level of the crista terminalis at the interface with the sinus venosus smooth myocardium. Myocardial anisotropy is also favored by a predominance of “end to end” gap junctions making transverse conduction up to 10 times slower than longitudinal conduction in that region (13).

Arenal et al. showed that transverse conduction block along the posterior sinus venosus is rate-dependent and the propensity to block depends on the location of the pacing site (sooner with posterior wall compared to lateral wall pacing) (5). This finding echoes the fact that pulmonary vein PACs frequently trigger atrial flutter (6), and therefore the sinus venosus will mostly be activated via the posterior wall of the right atrium in real life settings.

In our patient and with the pacing protocol we used, this conduction block occurred at shorter coupling intervals than the ones required for septal isthmus block. Nevertheless, this block appeared important for arrhythmia initiation and maintenance. By prolonging the conduction time required to go around the tricuspid valve, it allowed all cells along the circuit to exit their refractory period and transform unidirectional block into circular reentry.

### Generalizability of these observations

Our ability to map this phenomenon (septal isthmus block without immediate flutter induction) implies that the conduction time of the unidirectionally blocked beat around the tricuspid annulus was shorter than the refractory period of the septal isthmus. This is likely a rare finding, explaining why it could not be observed in 9/10 of the patients in this study. The observations of Arenal et al. (5) suggest that—contrary to what was seen in the presented patient—block of the posterior sinus venosus generally occurs prior to the septal isthmus block (for longer extra stimulus intervals). This contributes to prolonging the peri-tricuspid conduction time and immediate initiation flutter after septal isthmus block.

## Conclusions

The initiation of common atrial flutter takes place on the septal aspect of the cavo-tricuspid isthmus where functional unidirectional conduction block occurs. The direction of activation is therefore frequently counter-clockwise, and the arrhythmia stabilizes around anatomical and functional boundaries.

## Data Availability

The original contributions presented in the study are included in the article/Supplementary Material, further inquiries can be directed to the corresponding author.
