# Supplementary material for: Functional block in the initiation and maintenance of common flutter: detailed electrophysiological study and electro-anatomical mapping
Source: Front Cardiovasc Med. 2024 Nov 15;11:1494836. doi: 10.3389/fcvm.2024.1494836 (PMC11604714; doi:10.3389/fcvm.2024.1494836)

- S3 map (reference on coronary sinus S3)

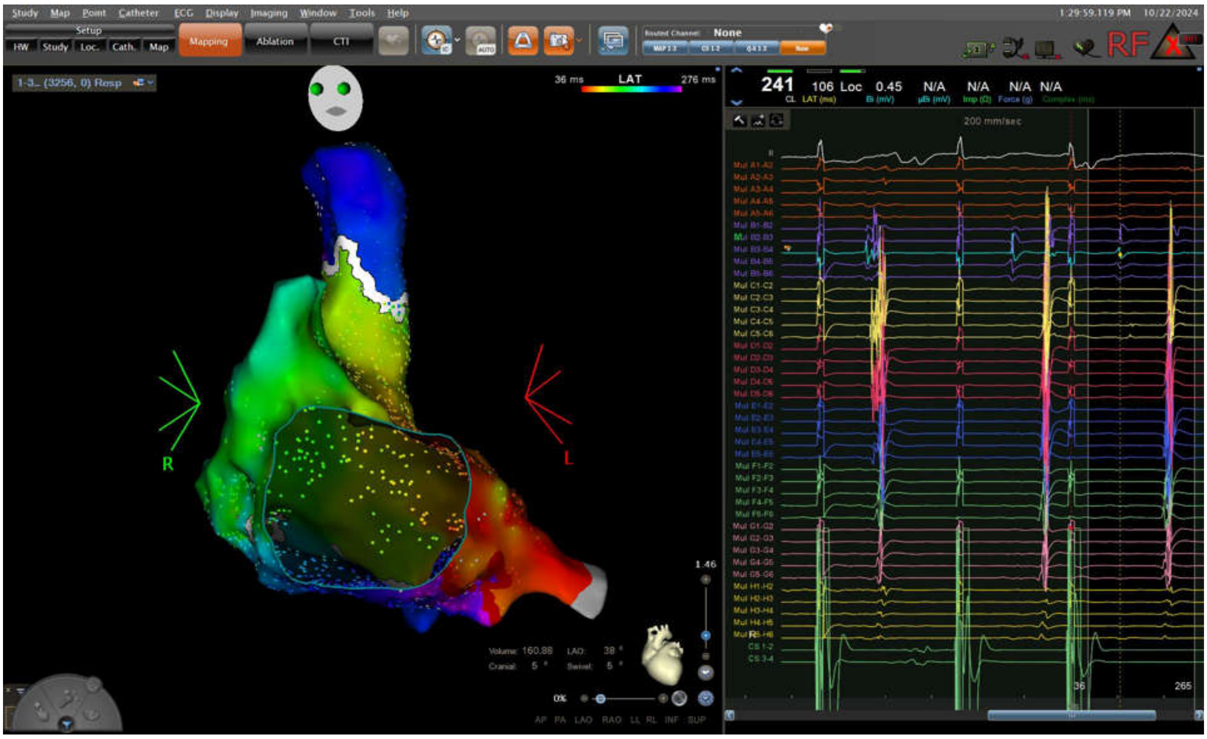

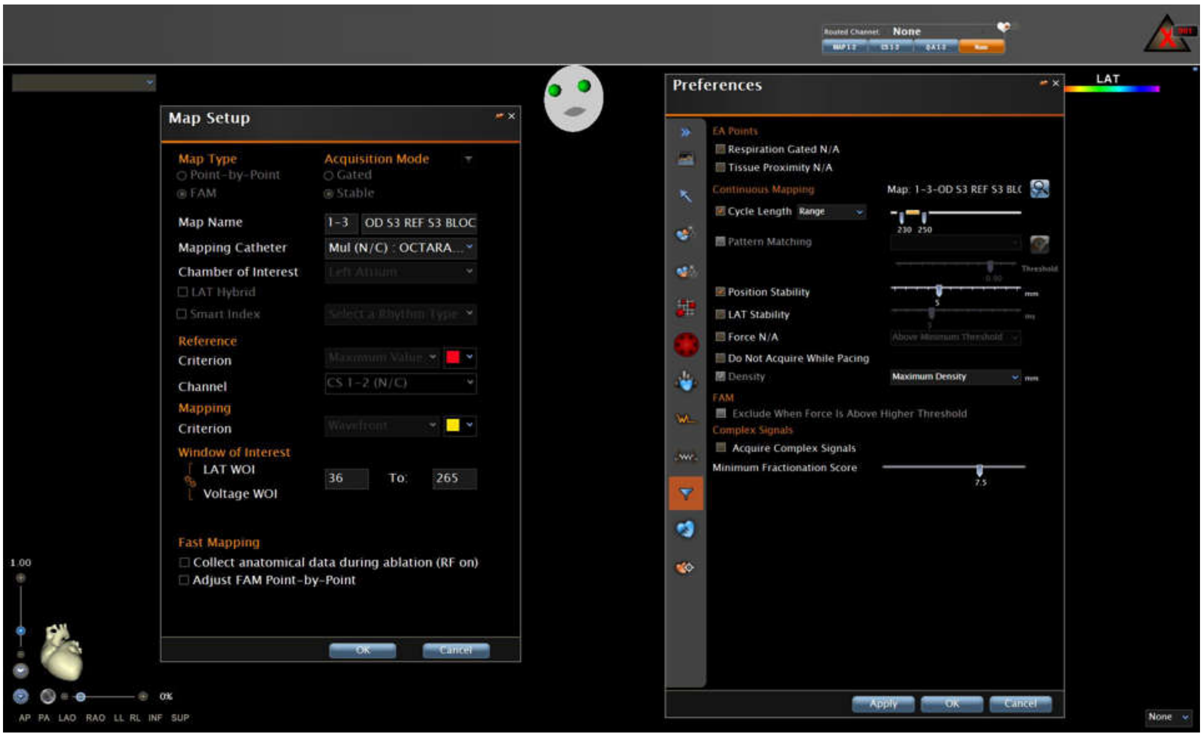

- S2 map (reference on coronary sinus S3)

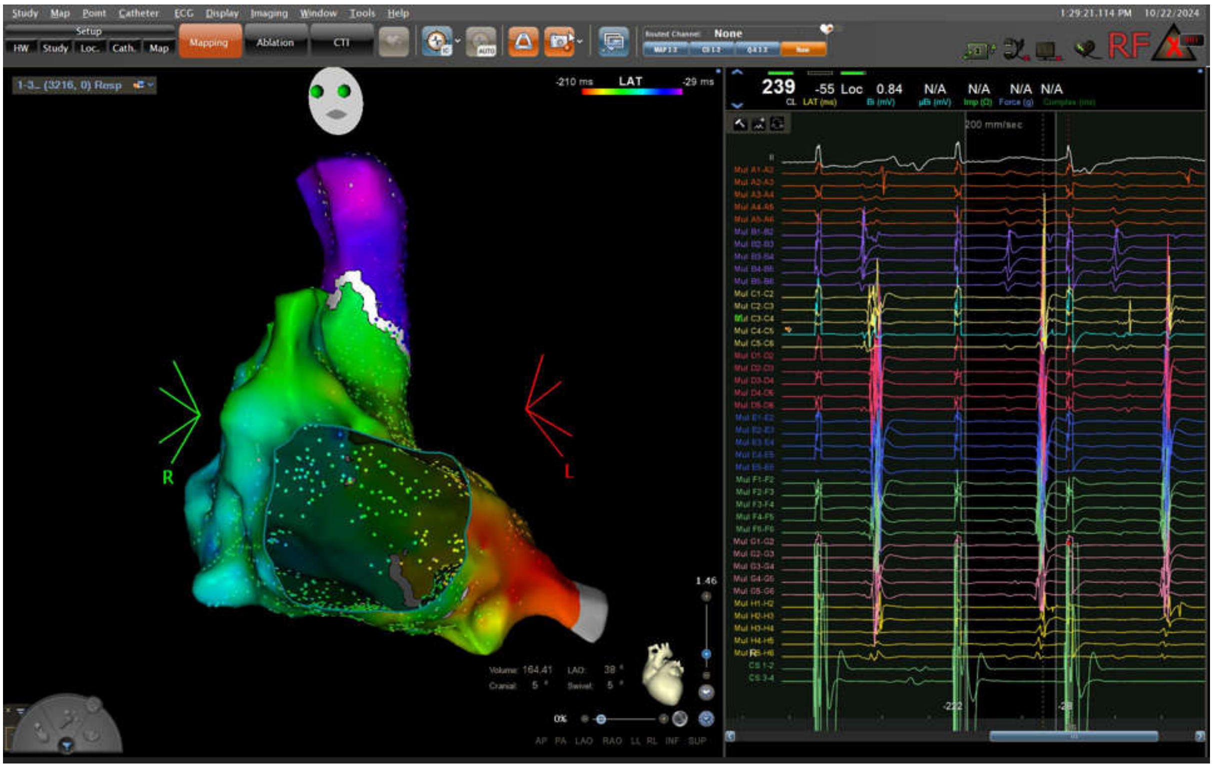

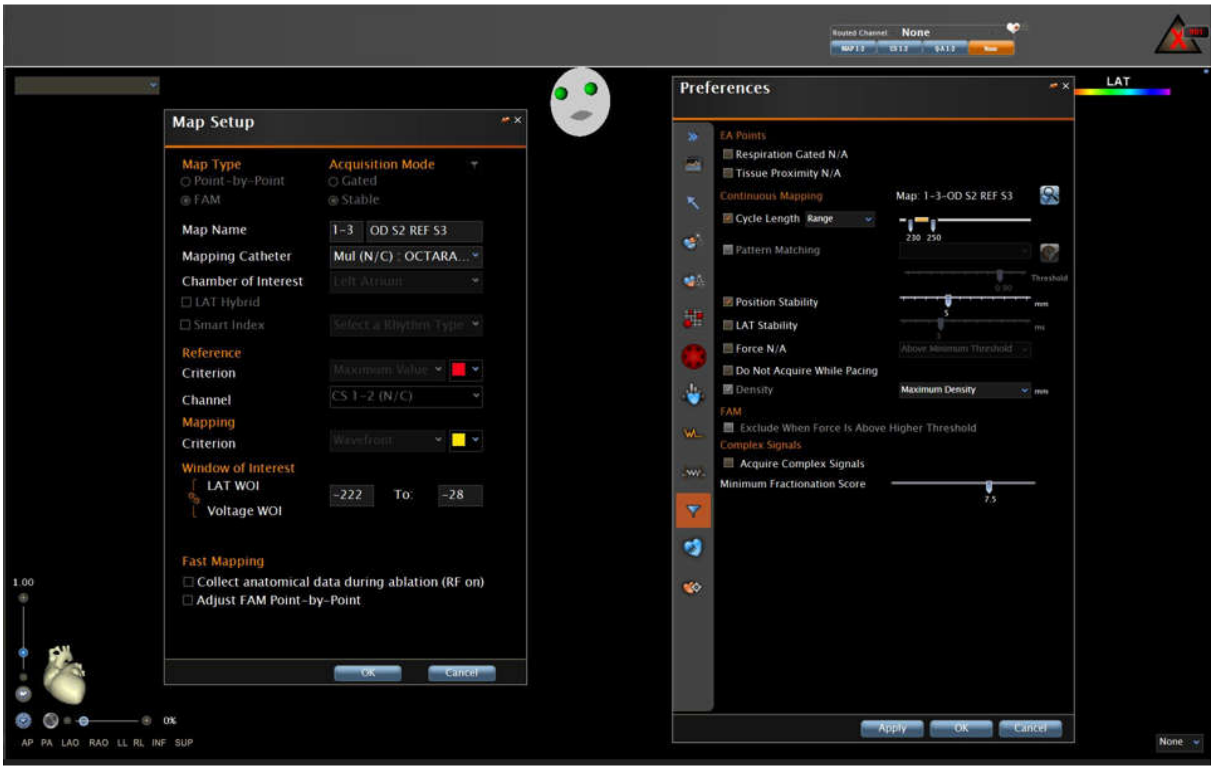

- S1 map (reference on coronary sinus S3)

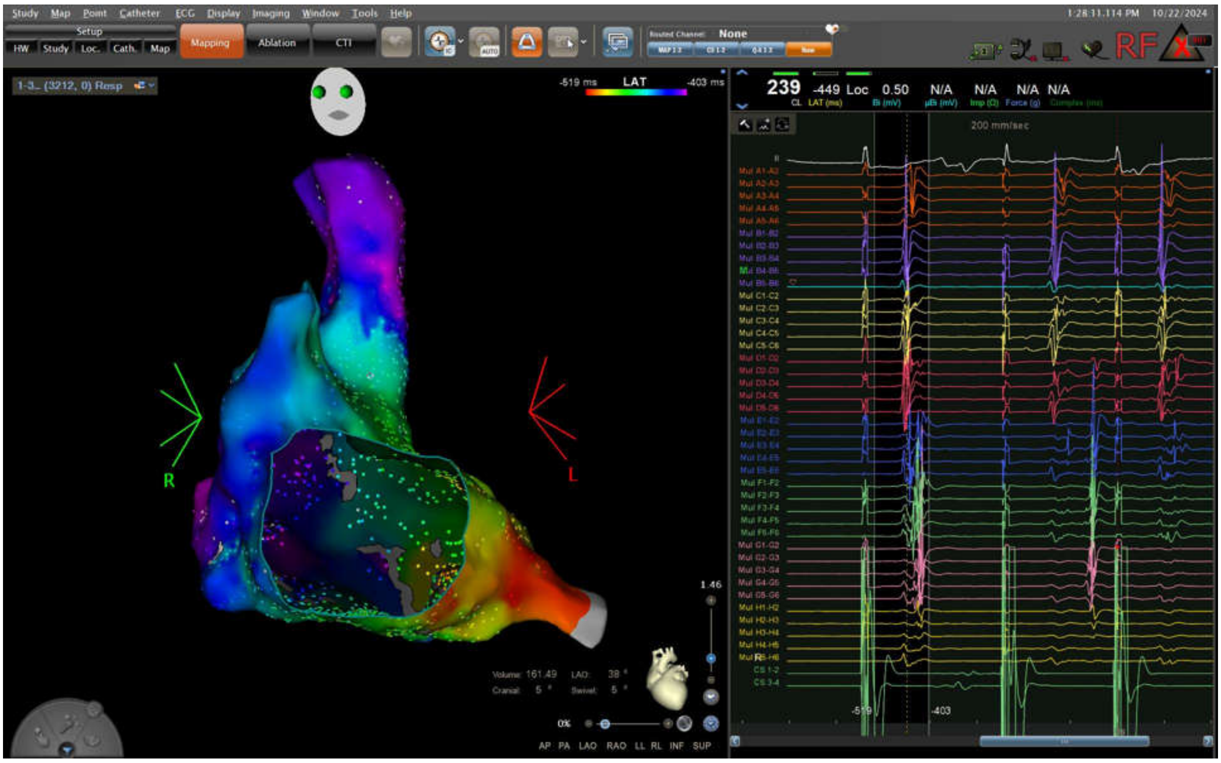

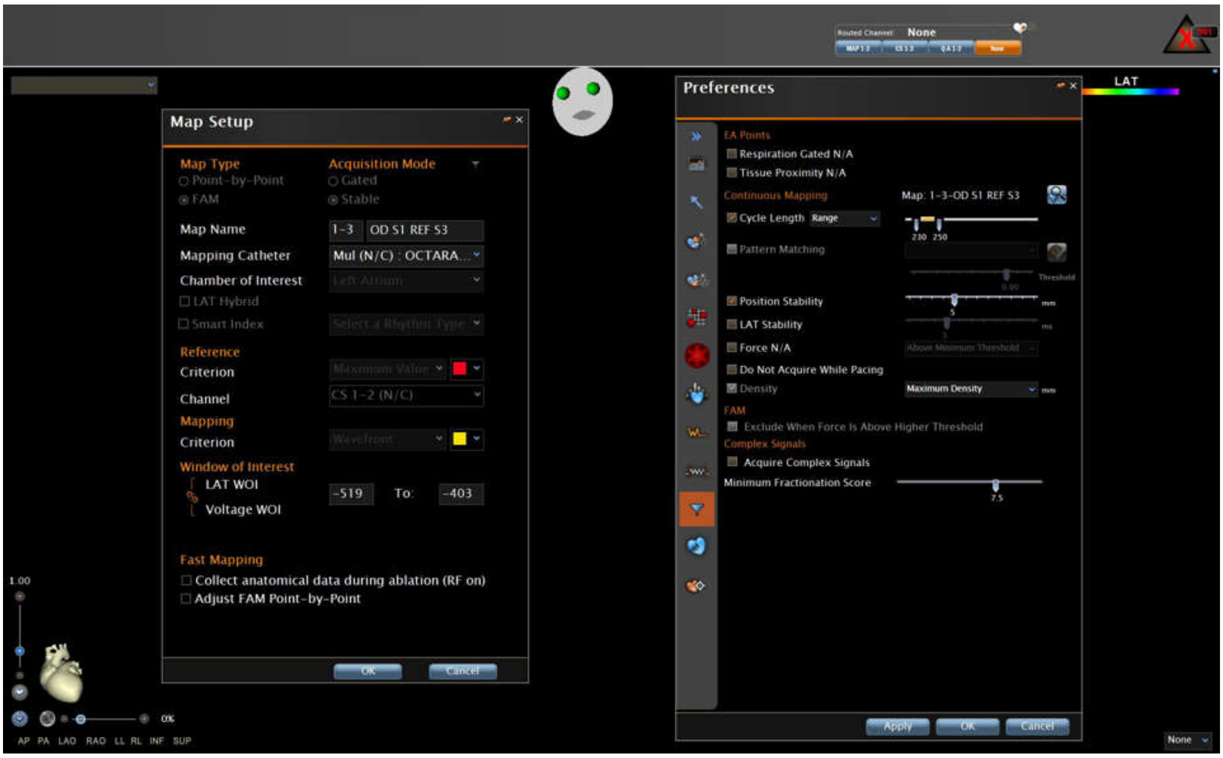

Supplement: Supplementary file 4 [file Datasheet1.pdf]
